# Regioregular narrow-bandgap-conjugated polymers for plastic electronics

**DOI:** 10.1038/ncomms14047

**Published:** 2017-03-28

**Authors:** Lei Ying, Fei Huang, Guillermo C. Bazan

**Affiliations:** 1 State Key Laboratory of Luminescent Materials and Devices, Institute of Polymer Optoelectronic Materials and Devices, South China University of Technology, Guangzhou 510640, China; 2 Department of Chemistry and Biochemistry, Center for Polymers and Organic Solids, University of California, Santa Barbara, California 93106, USA

## Abstract

Progress in the molecular design and processing protocols of semiconducting polymers has opened significant opportunities for the fabrication of low-cost plastic electronic devices. Recent studies indicate that field-effect transistors and organic solar cells fabricated using narrow-bandgap regioregular polymers with translational symmetries in the direction of the backbone vector often outperform those containing analogous regiorandom polymers. This review addresses the cutting edge of regioregularity chemistry, in particular how to control the spatial distribution in the molecular structures and how this order translates to more ordered bulk morphologies. The effect of regioregularity on charge transport and photovoltaic properties is also outlined.

## Abstract (web summary)

The continued search of high-performance semiconductors for organic electronics calls for smart molecular designs. Here, Ying *et al*. review the recent development of synthesizing regioregular narrow bandgap conjugated polymers and the effect of regioregularity on transistor and photovoltaic performance.

Conjugated polymers are characterized by an electronically delocalized *π*-conjugated backbone. This broad class of materials has attracted considerable attention from both industrial and academic research communities due to their potential to manufacture large-area and flexible organic electronic devices via low-cost solution processing techniques^1^,^2^,^3^,^4^. Remarkable progress has been achieved towards their integration into organic light-emitting diodes^5^,^6^,^7^,^8^, organic solar cells^9^,^10^,^11^,^12^, organic field-effect transistors (OFETs)^13^,^14^,^15^,^16^ and chemical or biological sensors^17^,^18^. A key consideration in the design of their overall molecular structure concerns how to optimize both the *π*-conjugated framework, relevant for optoelectronic function, and the side groups, which are needed for solubility, but which may also decrease morphological order in the solid state and thereby impact charge carrier transport. A delicate balance of the two structural components is required to achieve properties desirable for device function and a sufficiently high solubility for enabling straightforward processing options^19^,^20^.

Higher levels of morphological order in the solid state are more easily achieved when there is translational symmetry between repeat units, or substructures that comprise a number of repeat units, along the backbone vector^21^,^22^,^23^. When the repeat units in the backbone are asymmetric, which may be a result of the intrinsic geometry of the fragments that contribute to electronic delocalization or from the substitution pattern of the solubilizing groups, a fine control over their organization in the chain is needed, see Box 1. Indeed, the level of organization at the single chain level impacts the electronic coupling, secondary structure of the chain, solid-state microstructure and transport of the charge carriers in the solid state^24^.

Much of what has been learned on how regioregularity impacts the properties of semiconducting conjugated polymers has been through the classic studies of regioregular (RR) poly(3-hexylthiophene) (RR-P3HT), for which higher levels of crystallinity, red-shifted optical absorption^25^, larger charge carrier mobilities^26^ and ordered nanostructures^27^ are realized when the polymerization reaction introduces the asymmetric 3-hexylthiophene repeat unit in a strict head-to-tail (H–T) manner, see illustration a in Box 1. The presence of other configurations leads to materials that are less prone to crystallization. Furthermore, due to increased steric interference, tail-to-tail couplings decrease the coplanarity of adjacent thiophene heterocycles, and in doing so decrease intrachain electronic coupling. That RR-P3HT is more structurally homogeneous increases the persistence length, the nucleation density and crystallite size^28^.

Order across different length scales is often accompanied by improvements of properties relevant to electronic performance. Early work revealed that the chains of a self-assembled P3HT film with a regioregularity of 81% were preferentially oriented parallel to the substrate, whereas the sample with a regioregularity of 91% tended to exhibit lamellae perpendicular to the substrate^26^, and increased charge carrier mobility. For P3HT films with RR >90%, a distinct shoulder located on the long-wavelength side of the absorption maximum is observed as a result of closer interchain packing^29^. Studies of bulk-heterojunction films containing RR-P3HT and [6,6]-phenyl C_61_ butyric acid methyl ester (PC_61_BM) reported that the polymer lamellae preferentially orient orthogonal to the substrate, thus providing an increase in the optical anisotropy and a higher absorption of light. A solar cell device based on 95% RR-P3HT gave a power conversion efficiency (PCE) of ∼4%, much higher than the ∼1% obtained with a RR of 91% (ref. 25). However, it is worth pointing out that achieving the highest levels of structural precision in P3HTs may not be essential for reaching maximum PCEs^30^. Indeed, a comparison devices prepared using blends containing 86 or 96% RR-P3HT revealed that the lower RR material led to more thermally stable devices as a result of a decreased tendency to phase separate from the fullerene component^31^. These findings provide a glimpse of the challenges and importance of understanding how regioregularity at the molecular level extends to bulk properties and their role in triggering broad interest in the application of RR-P3HT in plastic electronics.

Extraordinary efforts have also been devoted toward developing semiconducting polymers with ‘donor–acceptor' type of architectures. These structures feature alternating electron-rich (donor, D) and electron-poor (acceptor, A) moieties along the backbone and this combination leads to optical bandgaps suitable for applications in organic solar cells^32^,^33^,^34^. As illustrated in Box 1, the internal organization of D–A copolymers should also be considered when the structural components lack a plane of symmetry perpendicular to the chain vector and the emerging evidence points to improved performance when random permutation of orientations are minimized in the polymer chain, particularly within the context of decreasing energetic disorder^35^. A range of complementary structural characterization techniques have therefore been applied to understand the origin of how structural precision leads to improved properties. These investigations provide an emerging understanding of how the relevant optoelectronic and morphological properties are modulated by the geometrical features of the polymer structures. Thus, it is timely to provide an overview of recently reported RR narrow-bandgap-conjugated polymers and their synthesis, together with studies on how molecular precision translates to increases of morphological order in the bulk and, ultimately, to more desirable properties when considering the fabrication of plastic electronic devices.

## Synthesis of RR polymers

Achieving well-defined structures begins with a consideration of the synthetic protocols. Here we provide a largely chronological account of how the challenges of regiochemistry have been overcome through advances in increasingly complex synthetic methods. Early contributions demonstrated the preparation of RR poly(3-alkylthiophene)s through transition metal catalysed methods^36^,^37^,^38^. A key synthetic feature is the selective metallation of dibrominated monomer precursors to generate 2-bromo-5-metalo-3-alkylthiophenes, which then react preferentially to produce H-T-arranged products. Indeed, the selectivity of these metallation reactions provides the key synthetic handle into achieving RR structures. The polymerization sequence is achieved by using transition metal initiators, such as Ni(dppp)Cl_2_, through a mechanism involving oxidative addition, transmetalation and reductive elimination. Under certain conditions, the reductive elimination step can be controlled to avoid detachment of the growing chain from the metal centre, thus leading to a chain-growth, living polymerization sequence^39^,^40^.

An alternative strategy to achieve structurally uniform backbones is to react two symmetrical monomer precursors in the polymerization reaction^41^; this approach is simpler mechanistically and removes any possibility of regioirregularity. Earlier studies^42^,^43^ demonstrated the polymerization of symmetric H–H and T–T dimers of 3-alkylthiophene, affording regiosymmetric polythiophenes. For the representative case of PBTTT, the polymerization is carried out based on two centrosymmetric comonomer units that naturally avoid formation of regiorandom fragments (see the illustration b in Box 1)^44^. Several other RR-conjugated copolymers have been developed using this approach^45^,^46^,^47^,^48^.

**Figure 1:**
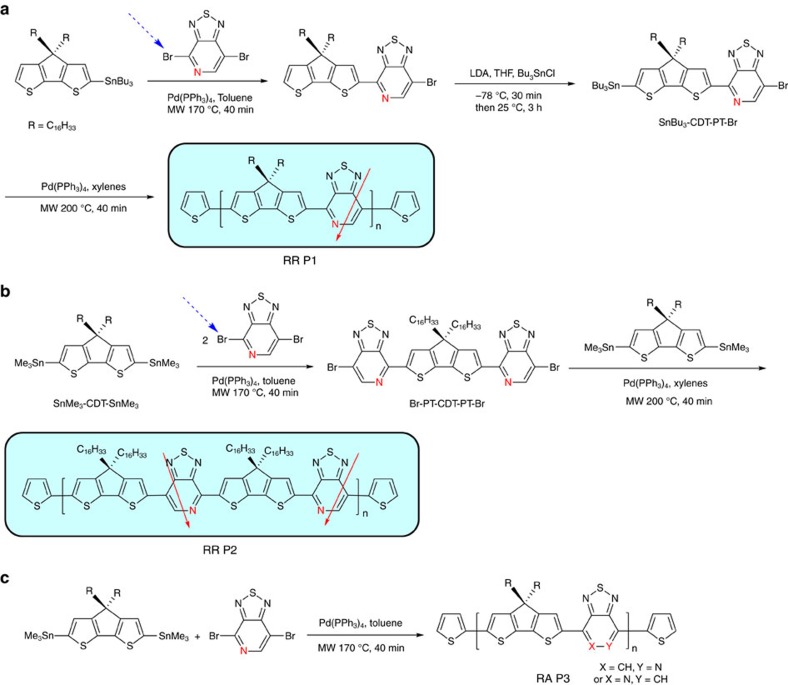
Synthetic strategy for conjugated polymers containing asymmetric PT moiety. (**a**) Synthetic routes of RR PT-based-conjugated polymers RR P1. (**b**) Synthetic routes of RR PT-based-conjugated polymers RR P2. (**c**) Synthetic route of regiorandom copolymer RA P3. The red arrows are a guide to highlight the orientation of the monomers relative to the backbone (Adapted, with permission, from ref. 66, copyright 2011 American Chemical Society).

While straightforward in design and practice, conventional metal-mediated cross-coupling polymerizations between asymmetric dihalide and *bis*-stannylated, or *bis*-boronic ester (acid), starting materials are typically not sufficiently regioselective. Achieving the target materials required developing original synthetic strategies in the case of the asymmetric PT building block^49^,^50^,^51^,^52^,^53^. As shown in Fig. 1, the reaction of 4,7-dibromo-[1,2,5]thiadiazolo[3,4-*c*]pyridine (PT-Br_2_) with mono- or *bis*-stannyled cyclopenta[2,1-*b*:3,4-*b′*]dithiophene (CDT) affords intermediates with precisely controlled orientation of N atoms. These units can be subsequently integrated into the corresponding polymers to generate RR structures. The basis for the specificity of the reaction can be traced to the reactivity of 2,5-dibromopyridine, for which the Pd-mediated cross-coupling of PT-Br_2_ with stannylated aromatic compounds was anticipated to occur preferentially at the C-Br adjacent to the pyridyl N atom^54^,^55^. This specific regioselective reaction can be used to produce two types of intermediates (Fig. 1) with strictly confined structures, which ultimately enabled a RR orientation of PT and CDT fragments along the backbone. Self-polymerization of the monomer Bu_3_Sn-CDT-PT-Br, containing the two complementary reactive functional groups, yields RR P1, in which all PT units are equally aligned relative to the backbone direction. The reaction of *bis*-stannyled CDT (CDT-Sn_2_) with the dibromo-macromonomer Br-PT-CDT-PT-Br provides RR P2 (also known as PCDTPT in the literature), where one finds that nearby PT units point in opposite directions. RA P3 corresponds to the polymer obtained by direct reaction of CDT-Sn_2_ with PT-Br_2_. Characterization by ^13^C NMR spectroscopy proved useful to confirm the proposed chemical structures, in particular, the chemical shifts of the carbon bridge of the CDT unit are determined by the orientations of adjacent PT units.

**Figure 2:**
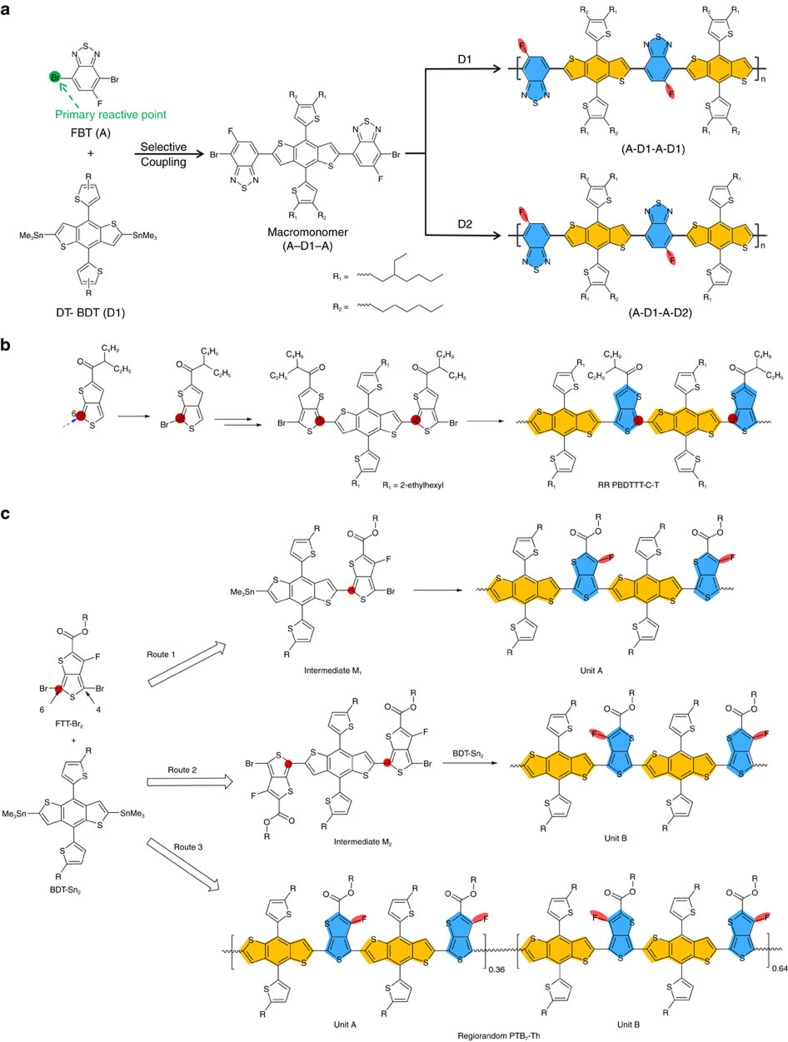
Representative examples using macromonomers as building blocks. (**a**) Synthetic route of RR A–D1–A–D1 and A–D1–A–D2 copolymers. The coloured ellipses highlight the different solubilizing groups. (Adapted, with permission, from ref. 56, copyright 2014 American Chemical Society). (**b**) Possible pathways for poly[[2,6′-4,8-di(5-ethylhexylthienyl)benzo[1,2-*b*;3,3-*b*]dithiophene][3-fluoro-2[(2-ethylhexyl)carbonyl]thieno[3,4-*b*]thiophenediyl]] (PTB7-Th) polymerization (Adapted, with permission, from ref. 60, copyright 2015 American Chemical Society). (**c**) Regio- and chemoselectivities of FTT-Br_2_ relevant for controlling polymerization products (Adapted, with permission, from ref. 61, copyright 2015 American Chemical Society).

5-Fluoro-2,1,3-benzothiadiazole (FBT) is an acceptor fragment with similar symmetry to that of PT. Moreover, dibromoFBT (4,7-dibromo-5-fluorobenzo[*c*][1,2,5]thiadiazole) exhibits different reactivities with respect to the two C-Br functionalities; a key point that enables access to RR FBT D–A polymers. As shown in Fig. 2a, the higher reactivity of the C-Br in the *meta*-position relative to fluorine can be used to generate an acceptor–donor–acceptor (A–D1–A) intermediate, described in the original report as a macromonomer, for which the fluorine atoms are distal to the electron-donating dithienyl-benzo[1,2-*b*:4,5-*b′*]dithiophene (DT-BDT) central unit^56^. Subsequent polymerization based on the macromonomer enables variations in the donor fragment substituents, thus affording polymers with unique A–D1–A–D1 or A–D1–A–D2 architectures that offer performance benefits over the random counterparts. The synthetic strategy in Fig. 2a provides entry into a wide range of polymers with tunable optoelectronic behaviour and solubility.

Poly(thieno[3,4-*b*]thiophene)benzothiophene (PTB)-based copolymers, which contain the thieno[3,4-*b*]thiophene (TT) moiety, are of particular interest for photovoltaic applications^57^,^58^,^59^. A range of regiochemistries would be reasonable to anticipate given the asymmetric nature of TT. Based on the monobromination at the 6-position of the TT (Fig. 2b) unit, and subsequent Stille coupling reaction, it is possible to obtain an intermediate comprising a TT-BDT-TT moiety, in which the sulfur atoms in both TT segments are positioned facing each other and are bridged by a BDT core^60^. This intermediate allows precise regiochemical control of the PBDTTT-C-T product. More to this point, a study emerged that systematically investigated the regio- and chemoselectivity of FTT-Br_2_, see Fig. 2c (ref. 61). Excellent selectivity for reactivity at the 6-position of FTT-Br_2_ was observed using a *bis-*stannylated co-reactant, which generates intermediate M1, and ultimately, unit A (Route 1), or intermediate M2 that can form unit B (Route 2). RR copolymers can be obtained by subsequent polymerization of separated M1 or M2 species. For comparison, the conventional one-pot polycondensation reaction using the dibromo-FTT and distannyled-BDT afforded a product with two distinctly different segments (unit A and unit B, Route 3).

**Figure 3:**
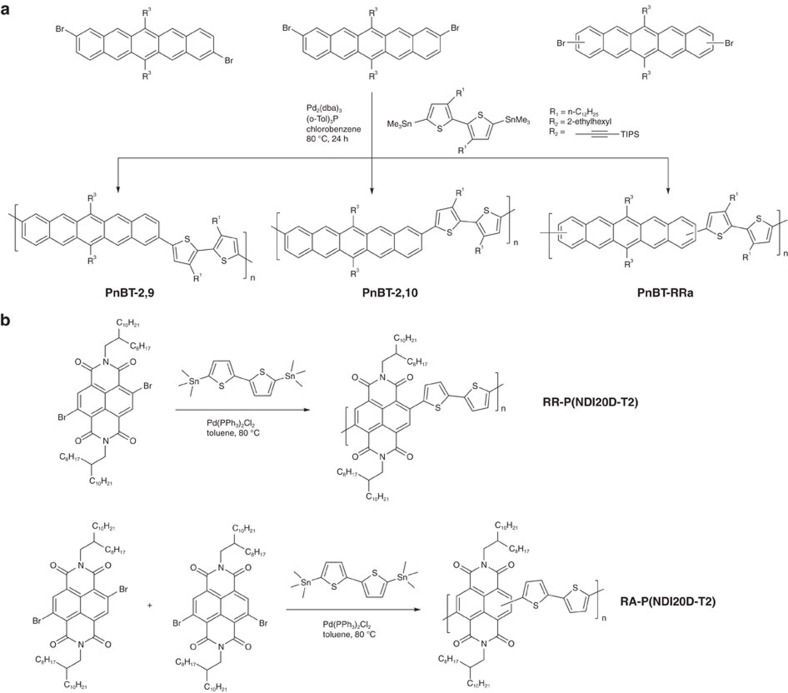
Established examples for control regioregularity of backbone. (**a**) Synthetic route of RR and RA copolymers containing pentacece (Adapted, with permission, from ref. 64, copyright 2012 The Royal Society of Chemistry). (**b**) Synthetic route of RR and RA copolymers containing NDI unit (Adapted, with permission, from ref. 65, copyright 2014 American Chemical Society).

Different substitution patterns can also give rise to structural imprecision. A representative case is provided by pentacene-containing copolymers, which are typically regiorandom^62^,^63^, due to difficulties in separating regio-isomeric dibrominated precursors. The copolymerization of pure 2,9- and 2,10-dibromopentacene derivatives with distannyled bithiophene comonomer gives the well-defined RR copolymers PnBT-2,9 and PnBT-2,10 (Fig. 3a)^64^. In contrast, mixed dibromopentace starting materials and the same bithiophene reactant yield the regiorandom counterpart PnBT-RRa. Control over regularity of the backbone structure has also been achieved for naphthalenediimide (NDI)-based copolymers (Fig. 3b)^65^. The copolymer RR-P(NDI2OD-T2) can be synthesized via the reaction of NDI2OD-2,6Br_2_ with 5,5′-*bis*(trimethylstannyl)-2,2′-dithiophene, while the regiorandom counterpart copolymer RA-P(NDI2OD-T2) can be obtained from the isomeric dibromide mixture of NDI2OD-2,6Br_2_ and NDI2OD-2,7Br_2_.

## Effects of regioregularity on charge carrier mobility

**Figure 4:**
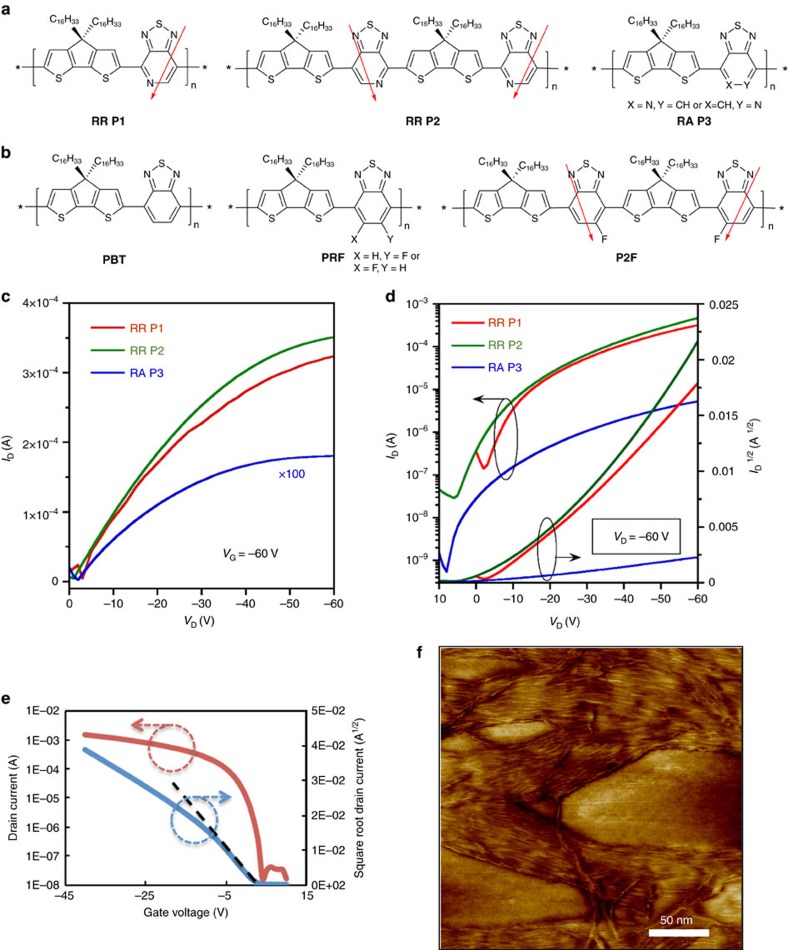
Representative regioregular-conjugated polymers for OFETs. (**a**) Molecular structures of RR and regiorandom PT-based copolymers. (**b**) Molecular structures of PBT, monofluorinated random PRF and regioregular P2F. (**c**,**d**) Output and transfer curves of spin-casted OFET devices (Adapted, with permission, from ref. 66, copyright 2011 American Chemical Society). (**e**) Transfer curve of RR P2 based on nano-grooved substrates (Reproduced, with permission, from ref. 72, copyright 2014 WILEY-VCH Verlag & Co. KGaA, Weinheim). (**f**) AFM image of RR P2 film. (Adapted, with permission, from ref. 72, copyright 2014 WILEY-VCH Verlag & Co. KGaA, Weinheim).

As mentioned previously, RR-P3HT established early on that levels of regioregularity modulate backbone conformations and relevant interchain stacking arrangements in the solid state^24^. Similar effects are also found in D–A type narrow-bandgap-conjugated polymers^66^. Consider, for example, the regio-isomeric copolymers consisting of alternating CDT and asymmetric PT (molecular structures shown in Fig. 4a)^66^. Compared with the regiorandom counterpart, OFETs prepared by spin coating the semiconductor from solution revealed that the RR copolymers with the PT in precisely defined orientations yielded a two orders increase in hole mobility, from 0.005 to 0.6 cm^2^ V^−1^ s^−1^ (Fig. 4c,d)^66^. These initial observations stimulated interest in understanding the structural origins of the improved charge carrier transport and led to the development of related polymer structures with similar regiochemical precision.

Considering the absence of noticeable differences between the optical and electrochemical properties of RR P2 and the regiorandom counterpart RA P3 for samples of similar molecular weights and dispersities, it is reasonable to attribute variations in hole mobility to different structural arrangements or orientations in the thin films. Indeed, grazing-incidence wide-angle X-ray scattering measurements of spin-casted films revealed that the regiorandom RA P3 forms crystallites arranged with a *π*–*π* stacking direction mainly perpendicular to the substrate, while in the RR P2 film, crystalline domains adopt a statistical mixture of *π*–*π* stacking orientations in the bulk^67^. Interestingly, the lamellar packing distance was observed to be shorter for RA P3 (∼2.5 nm) than for RR P2 (∼2.1 nm), a feature that was rationalized in terms of possible differences in the tilt angle of the backbone plane or side-chain conformations^67^. Despite that the orientation in the RR film deviates from the generally accepted preferred edge-on manner, such a mixture of ordered lamellar sheets of edge-on and face-on orientation may be beneficial for achieving charge carrier transportation in the three-dimensional network^68^,^69^. Near-edge X-ray absorption fine structure spectroscopy was also used to determine that the molecular orientation of RR P2 in blade-coated films depends on the nature of the underlying substrate^70^. The films have out-of-plane orientation where the backbones have a preferential ‘edge-on' alignment relative to the substrates surface, while the greatest degree of ‘in-plane' orientation occurs on the bottom side of a film deposited on a uniaxial nano-grooved substrate with increasing blade-coating rates. In this respect, the RR PT-based polymers provide an interesting platform for investigating how intermolecular stacking affects the charge carrier transport.

By combining nano-grooved substrates and a slow drying process, RR P2 can be macroscopically aligned into oriented crystalline fibres. Such highly orientated and ordered polymer chains achieved significant gains in charge mobility. Based on the fractionated sample with a molecular weight of 300 kDa, a hole mobility of 6.7 cm^2^ V^−1^ s^−1^ was reported, for which the transport was anisotropic with a higher mobility in the direction of the fibres^71^. Close inspection of the topside morphology of the 300 kDa RR P2 film on nano-grooved substrates was performed by high-resolution atomic force microscopy, as shown in Fig. 4f. The results show that the individual fibres are aligned within the bundles, leading to no obvious grain boundaries. AFM line-cut surface profile shows the width of an individual fibre to be ∼2–3 nm, which is comparable to the length of the repeat unit; thus the most reasonable way for polymer chains to align is in the direction of the long axis of the fibres. These features are consistent with long-range alignment of the semiconducting polymer chains, such that the transport occurs predominantly along the conjugated backbone with occasional *π*–*π* hopping to neighbouring chains.

It is worth pointing out that the mobility of >23 cm^2^ V^−1^ s^−1^ (and related higher values in the literature) for RR P2 in Fig. 4e is calculated in the low gate voltage (*V*_g_) regime (*|V*_g_*|*<20 V, dashed line) from devices in which the plot of *I*_d_^1/2^ (*I*_d_ is the drain current) versus *V*_g_ shows double slope characteristics^72^. If one calculates the mobility in the range between −25 and −35 V, one obtains a mobility of∼8 cm^2^ V^−1^ s^−1^. There is substantial lively debate in the literature regarding the physical basis responsible for the departure from the ideal metal oxide-semiconductor field-effect transistor model and its impact on accurate charge carrier mobility determination, as observed for both small molecule and polymer OFETs^73^,^74^. In the case of RR P2, examination of the device characteristics revealed that the double slope is likely due to electron trapping at the dielectric interface, which not only modifies the *I*_d_^1/2^ versus *V*_g_, but is also responsible for device variability through multiple scans^75^. Gate bias dependence of the contact resistance at the source and drain electrodes can also lead to non-idealities, as described in the literature^73^. Devices with characteristics such as those in Fig. 4e therefore need to be examined with care as they may lead to misassignment of carrier mobilities. Moreover, their unstable performance thus far precludes a practical technological impact.

Copolymers containing CDT and BT units (that is, PBT) exhibit relatively high-lying highest occupied molecular orbital levels (–5.0±0.2 eV), which are borderline for achieving long-term air stability (the highest occupied molecular orbital level is required to be below the air oxidation threshold of approximately –5.3 eV)^76^. To address this issue, the asymmetric monofluoro-substituted BT has been used as the acceptor, see Fig. 4b for molecular structures, which was anticipated to lower the orbital levels of the polymers and improve stability toward oxidation. Comparison of the RR copolymer (P2F) with precisely oriented fluorine atoms along the backbone revealed improved hole mobility (average 0.9±0.2 cm^2^ V^−1^ s^−1^) compared with the regiorandom counterpart (PRF, which has the asymmetric FBT units randomly oriented across the polymer backbone, average 0.3 cm^2^ V^−1^ s^−1^)^77^.

**Figure 5:**
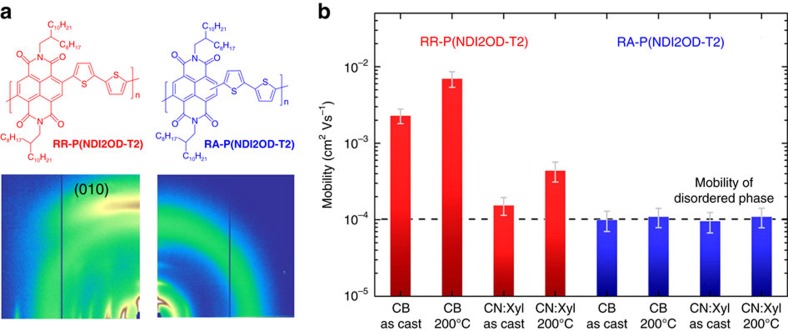
Effects of extension direction of conjugated polymer backbone. (**a**) Molecular structures and GIXD patterns of RR-P(NDIOD-T2) and RA-P(NDIOD-T2) thin films spin-cast from CB. (**b**) Comparison of vertical electron mobilities from films processed under different conditions (Adapted, with permission, from ref. 65, copyright 2011 American Chemical Society).

The effect of regularity on charge transport has also been demonstrated in polymers that differ in terms of the direction of conjugation extension, for example 2,6-/2,7-linked NDI frameworks^65^, 2,9-/2,10-linked pentacene^78^ or 1,6-/1,7-linked perylenediimides (PDI)^79^. As shown in Fig. 3b, a recent study reported a correlation between the backbone structure and the charge carrier mobilities of the NDI-based RR polymer RR-P(NDI2OD-T2), and its regiorandom counterpart RA-P(NDI2OD-T2) (also known as RI-P(NDI2OD-T2) in the literature), see Fig. 5 for molecular structures. Despite the greater than 100 nm shift in the optical bandgap in going from RR- to RA-P(NDI2OD-T2) in films coated from chlorobenzene (CB), these polymers exhibited comparable lowest unoccupied molecular orbital (LUMO) energy levels. This observation suggests that the optical shift is not caused by a further disruption of π-conjugation along the backbone of the RI polymer, which is understandable because regiorandom 2,6- and 2,7-NDI-thiophene linkages do not modify intramolecular steric demands. Grazing-incidence X-ray diffraction (GIXD) measurements indicated that the RR-P(NDI2OD-T2) film prefers a face-on arrangement, while the RA-copolymer film gave rise to a rather amorphous diffraction pattern, see Fig. 5a,b. By adjusting the film-forming conditions, RR-P(NDIOD-T2) films prepared from CB and chloronaphthalene:xylene solvent mixtures exhibited a similar crystalline structure along the three crystallographic axes, whereas thermal annealing led to larger vertical electron mobility. In contrast, a disordered phase was observed for RA-P(NDIOD-T2) films (Fig. 5b), while the ordered stacks along the lamellar direction did not show detectable *π*-stacking. Although thermal annealing leads to considerable increase in the coherence length and degree of crystallinity in the lamellar direction, the vertical mobility remains nearly unchanged (Fig. 5b). Moreover, from Fig. 5b, one observes that the RR copolymer exhibits higher vertical electron mobility than the random counterpart.

Pentacene-based copolymers constructed via attachments at the 2,9- or 2,10-positons are also worth considering within the context of structural precision^78^. A synthetic route to obtain pure 2,9- and 2,10-dibromopentacene precursors was reported, see Fig. 3a, which enabled the synthesis of regioregular pentacene-containing copolymers^80^. Despite the relatively low n-type mobility (10^−4^–10^−5^ cm^2^ V^−1^ s^−1^) of the resulting RR copolymers, these values are considerably higher than what is obtained when using the regiorandom counterpart (8 × 10^−7^ cm^2^ V^−1^ s^−1^). RR pentacene-based conjugated polymers consisting of alkylated-bithiophene as the comonomers were examined, please refer to Fig. 3a for their molecular structures. Charge carrier mobility measurements showed that the copolymers based on RR PnBT-2,10 (Fig. 3a) and the regiorandom counterpart had similar hole mobility in the order of 10^−4^∼10^−3^ cm^2^ V^−1^ s^−1^, while the hole mobility of about 0.03 cm^2^ V^−1^ s^−1^ that obtained from optimized device fabrication conditions based on RR PnBT-2,9 (Fig. 3a) was much higher. Compared with PnBT-2,10, which has a bent ‘zigzag' backbone, PnBT-2,9 has a more linear and rod-like structure and more easily forms ordered domains for better charge transport. GIXD measurements indeed revealed slightly shorter lamellar spacing of 14.5 Å for PnBT-2,9 than that of 15.8 Å for the other two copolymers^64^. These findings provide a relevant metric on how controlling the connectivity for modulating electronic delocalization impacts the thin film order and thereby electronic properties.

## Effect of regioregularity on photovoltaic performance

**Figure 6:**
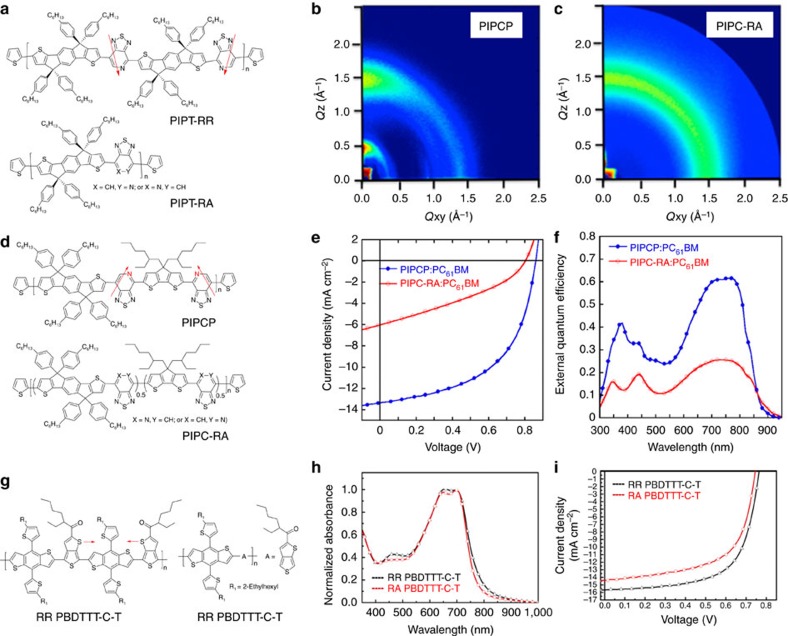
Representative regioregular-conjugated polymers for polymer solar cells. (**a**) Chemical structures of PIPT-RR and PIPT-RA. (**b**) Grazing-incidence wide-angle X-ray scattering (GIWAXS) patterns of PIPCP thin film. (**c**) GIWAXS pattern of PIPC-RA thin films. (**d**) Chemical structures of PIPCP and PIPC-RA (Adapted, with permission, from ref. 84, copyright 2014 American Chemical Society). (**e**,**f**) *J*–*V* characteristics and external quantum efficiency spectra of polymer solar cells based on PIPCP:PC_61_BM and PIPC-RA:PC_61_BM. (**g**) Chemical structures of RR PBDTTT-C-T and RA PBDTTT-C-T (Reproduced, with permission, from ref. 84, copyright 2014 American Chemical Society). (**h**) Ultraviolet–vis absorption spectra of RR PBDTTT-C-T and RA PBDTTT-C-T thin films. (**i**) *J*–*V* characteristics of solar cells based on RR PBDTTT-C-T and RA PBDTTT-C-T (Adapted, with permission, from ref. 60, copyright 2015 American Chemical Society).

Remarkable progress with the certified power conversion efficiency up to 11.5% has been achieved for the application of narrow-bandgap-conjugated polymers for organic solar cells^81^. As illustrated in the preceding examples, controlling the organizational precision within D–A copolymers leads to improved performance factors when included as the OFET semiconductor layer. It stands to reason to examine to what extent these trends extend to the self-organization of bulk-heterojunction (BHJ) active layers, in which the D–A copolymer behaves as the p-type component and the fullerene derivatives provide the complementary n-type phase. One consideration is that random permutations of asymmetric units open the possibility of energetic disorder^35^ that may localize the charge carrier wave functions and in this way influence charge extraction and recombination. The orientation of asymmetric species also affects the molecular configurations and consequently the self-organization of the blend films. Yet another consideration is that a greater driving force for crystallization for a structurally homogenous polymer can lead to modification of phase separation during the timescale of film evolution. With regard to these effects, the regioregularity of conjugated polymers can affect the current density and fill factor, and thus the overall photovoltaic performance. In addition, as the open-circuit voltage (*V*_OC_) of polymer solar cells is primarily determined by the tail of the density of states^82^, the reduced disorder in such RR narrow-bandgap-conjugated polymers offers a platform to study whether structural disorder of the polymer backbone can result in the loss of *V*_OC_. To address these issues, an initial investigation of the effects of regiochemistry control on photovoltaic performance was performed using the RR-conjugated polymer PIPT-RR (also known as PIPT-RG in the literature^83^, molecular structures shown in Fig. 6a), which contains PT and indacenodithiophene (IDT) units. In PIPT-RG, the nitrogen atoms in the PT unit are precisely arranged along the backbone so that each one has an adjacent proximal and an adjacent distal counterpart across the two IDT flanking units. Despite a lack of obvious differences in the orbital energy levels and optical bandgaps, the higher charge carrier mobility of the PIPT-RR relative to PIPT-RA improved the photovoltaic performance of the polymer solar cells, compared with the regiorandom counterpart in terms of higher *V*_OC_ both in conventional and inverted device configurations^83^. These findings were amongst the first to highlight the benefits of controlling the regiochemistry of PT-containing narrow-bandgap-conjugated polymers.

The RR narrow-bandgap-conjugated terpolymer PIPCP, and its less structurally precise counterpart PIPC-RA (molecular structures shown in Fig. 6d) have also been constructed by combining two different donor fragments (IDT and CDT), and forcing the pyridyl N-atoms to point towards the CDT unit^84^. Optical spectroscopy reveals an absorption profile of PIPCP that is red-shifted, narrower in width, and exhibits more pronounced vibronic-like features, relative to PIPCP-RA. Grazing-incidence wide-angle X-ray scattering measurements of PIPCP show a strong reflection peak preferentially aligned in the out-of-plane direction at *q*=1.47 Å^−1^ that corresponds to *π*–*π* stacking, and an intense reflection at *q*=0.24 Å^−1^, which is assigned to the alkyl chain organization. Both signals extend to the in-plane direction, suggesting that both face-on and edge-on orientations exist in the thin film (Fig. 6b). In contrast, the regiorandom counterpart PIPC-RA shows a higher contribution from an amorphous-like ring, instead of clear peaks (Fig. 6c). This structural insight indicates that the molecular precision of PIPCP translates into BHJ films with higher levels of morphological order in the donor phase. Figure 6e,f shows the current density–voltage characteristics (*J*–*V*) and the external quantum efficiency spectra of the solar cells, one finds considerable improvements with the regioregular structure. This increase in PCE is attributable to the increased current density.

Of particular relevance is the low photon energy loss (*E*_loss_). *E*_loss_ is defined here as *E*_g_−e*V*_OC_, where *E*_g_ is the optical bandgap of the donor polymer, and e*V*_OC_ is obtained from BHJ blends prepared with PIPCP and either PC_61_BM or [6,6]-phenyl C_71_ butyric acid methyl ester (PC_71_BM). The *E*_loss_ value of the PIPCP blends was measured to be 0.52±0.02 eV (ref. 85), which is lower than the majority of narrow-bandgap-conjugated polymer reported in the literature, and is lower than the widely referenced 0.6 eV limit. Subsequent studies revealed that the energy of the bandgap and the charge transfer state was nearly equal, suggesting that PIPCP:PC_61_BM blends provide minimal energy losses when considering the exciton and the charge transfer state, except for the relatively small offset between the tail state of the LUMO levels of the donor and acceptors. Of particular interest is that PIPCP has a low Urbach energy (*E*_U_), which describes the widths of density of state tails in the case of disordered semiconductors, at about 27 meV (ref. 85), close to thermal energy (*kT*=25 meV) and the disorder-free indacenodithiophene-co-benzothiadiazole (IDTBT) copolymer (*E*_U_=24 meV)^86^. The high morphological order, low Urbach energy, and low energetic disorder indicate that reducing disorder (energetic and morphological) can minimize voltage losses, as proposed by a recent theoretical contribution^87^.

Despite that PTB based copolymers show excellent PCEs of about 10%, the backbone structures remain relatively undefined due to the asymmetric TT moiety. To address this issue, the RR polymer of PBDTTT-C-T was developed by controlling the orientation of sulfur atoms in the intermediate, with the molecular structures shown in Fig. 6g. Compared with the random counterpart, the RR copolymer shows a narrower optical bandgap (Fig. 6h), higher crystallinity, higher hole mobility, and more uniform morphology with better interpenetrating networks in the blend films. Inverted BHJ solar cells based on RR PBDTTT-C-T exhibit a PCE that is 19% higher than what is observed for the regiorandom counterpart, particularly as a result of increased current density and fill factor (Fig. 6i). The enhanced PCE for RR PBDTTT-C-T presumably correlates with the improved absorption and increased charge carrier mobility as a result of effective ordering between polymer chains^60^. Similar observations have been reported for the recently developed RR copolymer PBDT-TSR that comprises the asymmetric TT unit^88^. Although the orbital energy levels and miscibility with PC_71_BM were not greatly influenced by the backbone configuration, the RR copolymer yielded a PCE of >10%, which was higher than the random counterpart. In addition to the asymmetric TT unit of the PTB series polymers, researchers investigated the symmetry of donor comonomer by constructing thienothiophene quinoidal character with enlarged molecular size^89^. It is worth noting that copolymers with symmetric donor comonomer exhibit comparable PCE at the same order of PTB7, while the copolymer based on the comonomer that only has C1 symmetry exhibits extremely low PCE. These findings further highlight the importance of molecular structure control for attaining high performance.

PDI-based conjugated polymers exhibit regioregularity considerations similar to those mentioned for NDI-containing systems. Indeed, strategic design led to the preparations of the regioregular PDI-based copolymer RR PDI-diTh and the regiorandom counterpart RA PDI-diTh (also known as r-PDI-diTh and i-PDI-diTh in the literature, respectively)^90^. The regioregular copolymer RR PDI-diTh exhibits improved photovoltaic performance with respect to the RA PDI-diTh, due to the higher current density of the former^90^.

**Figure 7:**
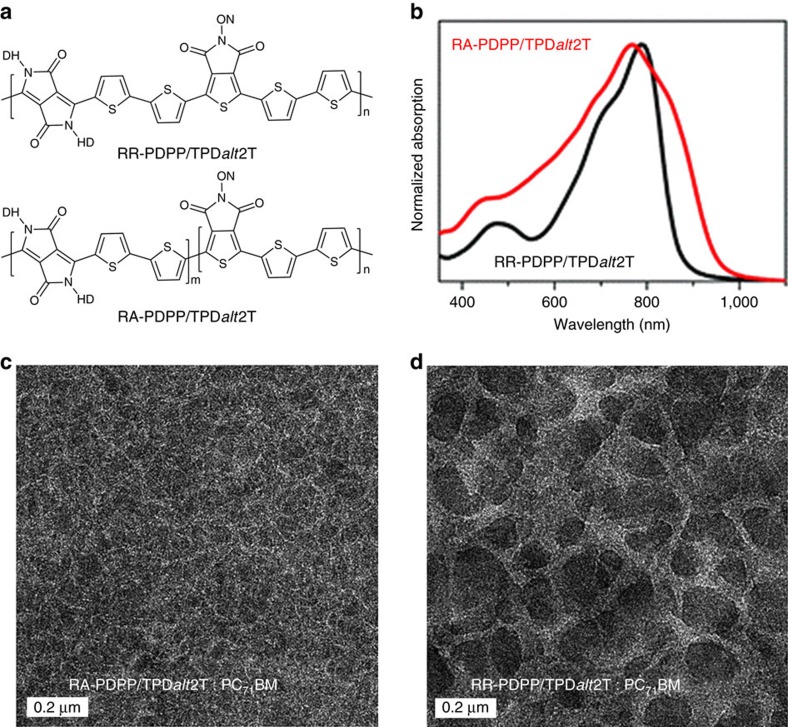
Effects of regioregular architecture on polymer solar cells. (**a**) Molecular structures of RR-PDPP/TPD*alt*2T and RA-PDPP/TPD*alt*2T. (**b**) Ultraviolet–vis absorption of pure polymer films. (**c**,**d**) Transmission electron microscopy (TEM) images of polymer:PC_71_BM blend films. DH=2-hexadecyl, ON=1-octylnonyl (Adapted, with permission, from ref. 91, copyright 2014 The Royal Society of Chemistry).

As a final illustrative example, FBT RR copolymers also yield improved PCEs relative to their regiorandom analogues^56^. The primary source of improvement was the increased short-circuit current. Furthermore, isomeric RR and random conjugated copolymers have been reported that comprise multi-components of diketopyrrolopyrrole, thienopyrrolodione, and bithiophene units (molecular structures shown in Fig. 7a)^91^. Comparison of the RR polymer RR-PDPP/TPD*alt*2T (also known as reg-PDPP/TPD*alt*2T in the literature^91^) and its regiorandom counterpart RA-PDPP/TPD*alt*2T (also known as ran-PDPP/TPD*alt*2T in the literature^91^) revealed that the statistical distribution of monomeric units had a pronounced influence on the optical bandgaps and molecular orbital energy levels. Although the absorption profile of the random polymer broadened towards a longer wavelength (Fig. 7b), the decreased LUMO energy level associated with the coarser morphology (as shown in the transmission electron microscopy (TEM) images in Fig. 7c,d) and a rougher surface led to lower short-circuit current density in the random polymer^91^. There is therefore ample evidence from many research groups that indicate positive improvements in critical device variables through controlling the regiochemistry of narrow-bandgap-conjugated polymers, and related structures.

## Outlook and perspective

Precise control over the orientation of asymmetric units along the backbone enables the achievement of regioregular narrow-bandgap-conjugated polymers. These materials exhibit advantageous optoelectronic and morphological properties relative to their regiorandom derivatives, and these competitive advantages should be kept in mind when considering asymmetric building blocks, such as 5*H*-dithieno[3,2-*b*:2′,3′-*d*]pyran^92^, thieno-benzo-isoindigo^93^, and diketopyrrolopyrrole derivatives that functionalized with aromatic units^94^. Understanding why the integration of regioregular structures into field-effect transistors leads to higher charge carrier mobilities requires a full theoretical understanding of the transport mechanism, including the effects of the intramolecular dipole orientations, the electronic density of states, the general morphological distribution of polymer chains, and supramolecular self-organization in a well-controlled macroscopic alignment of polymer chains. Truly delocalized electron transport in a polymer chain may be attained^95^, which has the potential to elucidate the intrinsic limits of the charge carrier mobility in soft semiconducting matter. The significantly enhanced photovoltaic performances of regioregular donor–acceptor narrow-bandgap polymers highlight the need for precise control over the distribution of monomeric units, where increased order can reduce open-circuit voltage losses, and provide a more favourable self-organization of the BHJ films.

To provide a general guideline for the design of RR narrow-bandgap polymers for solar cell applications, more fundamental knowledge is required concerning the effects of regioregularity on electronic structures, photochemical stabilities, the exciton dissociation dynamics of the charge carrier process, and more importantly, the correlation between the energy of the different states and specific morphological characteristics. Various thin film characterization techniques such as grazing-incidence X-ray diffraction, resonant soft X-ray scattering, transmission electron microscopy, and tomographic techniques can be used to probe nanoscale and intermolecular organization features, and the evolution of the crystallite nucleation and growth during the film casting procedure.

## Additional information

**How to cite this article:** Ying, L. *et al*. Regioregular narrow-bandgap-conjugated polymers for plastic electronics. *Nat. Commun.* **8,** 14047 doi: 10.1038/ncomms14047 (2017).

**Publisher's note:** Springer Nature remains neutral with regard to jurisdictional claims in published maps and institutional affiliations.

## Overview of regioregular-conjugated polymers.

Regioregular (RR)-conjugated polymers follow a strict orientation of alternating asymmetric repeating units throughout the polymer backbones; in other words, they are described by translational symmetry along the backbone vector. The most representative case is poly(3-alkylthiophene) (P3AT), which has three possible connections, head-to-tail (H–T), head-to-head (H–H) or tail-to-tail (T–T), between two 3-alkylthiophene repeat units (**a**). RR P3AT has a strict H–T manner, while regiorandom P3AT contain a mixture of three connections. In this case, the regioregularity of P3ATs is denoted as the percentage of the arranged H–T units in the backbone.

In addition to fixing the H–T connections in P3AT structures, an alternative strategy for constructing symmetric thiophene-containing copolymers is to incorporate a *π*-conjugated spacer between H–H coupled 3-alkyl substituted subunits^41^. A representative example of this type of regiosymmetric polymer is the poly(2,5-*bis*(3-alkylthiophen-2-yl)thieno[3,2-*b*]thiophene) (PBTTT), which can be prepared from two symmetrical monomers as shown in the above scheme (**b**)^44^. The decreased density of alkyl chains in PBTTT with regard to RR-P3ATs allows for greater side-chain interdigitation and closer intermolecular *π*–π distances that promote self-organization and facilitate high charge carrier mobility.

D–A polymers are also subject to regioregularity considerations when either (or both) of the D or the A moiety is (are) asymmetric. One case in point is the [1,2,5]thiadiazolo[3,4-*c*]pyridine (also known as pyridyl[2,1,3]thiadiazole, PT) acceptor heterocycle, for which a carbon (C) atom in the widely used benzo[2,1,3]thiadiazole (BT) fragment is replaced by a nitrogen (N) atom^49^,^50^. This PT for BT modification results in a backbone with higher electron affinity and leads to a narrower optical bandgap, at the expense of reducing the symmetry^51^,^52^,^53^. The above illustration (**c**) shows two generic RR-conjugated polymer systems in which the asymmetric PT fragment is oriented in specific orientations relative to the backbone^66^. In these molecular structures, ‘Ar' is a generic, and most typically electron rich, comonomer unit that links the PT units in conjugation, and which can be selected to fine tune optical properties, the promotion of crystalline domains, and/or large charge carrier mobility values. In type I, the PT is positioned along the chain vector so that the pyridyl nitrogens point in same direction, whereas in type II the PT heterocycles alternate in orientation. Of particular interest is that the hole mobility of copolymers with both type I and II RR structures are approximately two orders of magnitude higher than that of their regiorandom counterpart for spun cast films^66^ and higher mobilities can be accessed by specifically tailored film processing techniques^67^. (Adapted, with permission, from ref. 66, copyright 2011 American Chemical Society).
